# Activation-induced cytidine deaminase overexpression in double-hit lymphoma: potential target for novel anticancer therapy

**DOI:** 10.1038/s41598-020-71058-y

**Published:** 2020-08-25

**Authors:** Jingcheng Zhang, Yifen Shi, Mingzhe Zhao, Huixian Hu, He Huang

**Affiliations:** 1 Bone Marrow Transplantation Center, The First Affiliated Hospital, Zhejiang University School of Medicine, Hangzhou, 310003 Zhejian People’s Republic of China; 2 Department of Hematology, Jinhua Hospital of Zhejiang University (Jinhua Municipal Central Hospital), Jinhua, 321100 Zhejiang People’s Republic of China; 3 Department of Hematology, The First Affiliated Hospital of Wenzhou Medical University, Wenzhou, 325000 People’s Republic of China

**Keywords:** Biological techniques, Cancer, Oncology

## Abstract

Activation-induced cytidine deaminase (AID) is one kind of the mutant enzymes, which target regulating the immunoglobulin (Ig) gene in Burkitt’s lymphoma to initiate class switch recombination (CSR), resulting in c-Myc chromosomal translocation. However, it is not clear that whether AID induces c-Myc/IgH translocation in double-hit lymphoma (DHL) with c-Myc gene translocation. In this study, the AID in DHL tissues and classical diffuse large b-cell lymphoma (DLBCL) tissues were compared. The results suggested that AID is of important value in predicting DHL, stronger CSR of AID was observed in DHL patients, which exhibited AID overexpression and c-Myc gene translocation of DHL after CSR induction. It is concluded that AID directly induces CSR in DHL and may result in c-Myc gene translocation. Targeting AID may be a good treatment regimen for DHL.

## Introduction

Diffuse large B-cell lymphoma (DLBCL) is the most common non-Hodgkin lymphoma with a high degree of heterogeneity in clinical and biological behavior and a poor prognosis in high-risk patients. With the development of molecular biology in recent years, 6–14% DLBCL have been found c-Myc gene translocation that often accompanied with bcl-2 or bcl-6 translocation, known as double-hit lymphoma (DHL)^1^. The CR (complete response) to the traditional R-CHOP (rituximab, cyclophosphamide, doxorubicin, vincristine, prednisone) regimen for DHL is only about 20%. Currently, most of DHL adopts the strong chemotherapy regimen combined with autologous hematopoietic stem cell transplantation^2,3^, which however haven’t benefit the treatment of DHL, reported by a multi-center comparative study^4^.

Most DHL arise from germinal centers, which are the main sites where B cells switch the class of their antibodies (CSR) in response to antigenic stimulation^5^. Activation-induced cytidine deaminase (AID) in the germinal center induce the high mutation degree of B cells, which produce different-encoded antibody B cells to complete CSR, ultimately leading to the occurrence of proto-cancerous chromosomal translocation between immunoglobulin c-Myc and IgH in B cells^6,7^. Several studies have shown that AID expression in DLBCL has a poor prognosis^8,9^.

AID is expressed in various types of b-cell lymphoma, including follicular lymphoma, Burkitt’s lymphoma^10,11^, mucosa-associated lymphoid tissue lymphoma, and chronic lymphocytic leukemia^12–14^. However, it has not been proved that AID leads to increased CSR activity and c-Myc gene translocation in DHL in previous studies. The prognostic value of AID expression in DHL is still controversial probably due to lack of evidence.

In this study, immunohistochemistry and Western Blot were used to detect and analyze the AID expression in 20 DHL and 20 classic DLBCL tissues respectively. The relation between AID expression and clinicopathological parameters as well as prognosis were established and its application value was explored. Besides, the CSR of patients with DHL and classical DLBCL respectively were analyzed by detecting the immunoglobulin in the peripheral blood of patients. The influence of AID on CSR level and c-Myc translocation in DHL was analyzed by cell experiment. The results of this study will provide reference for clinical diagnosis, prognosis and molecular targeted therapy of DHL.

## Results

### AID protein overexpression in patients with LDH

**Figure 1:**
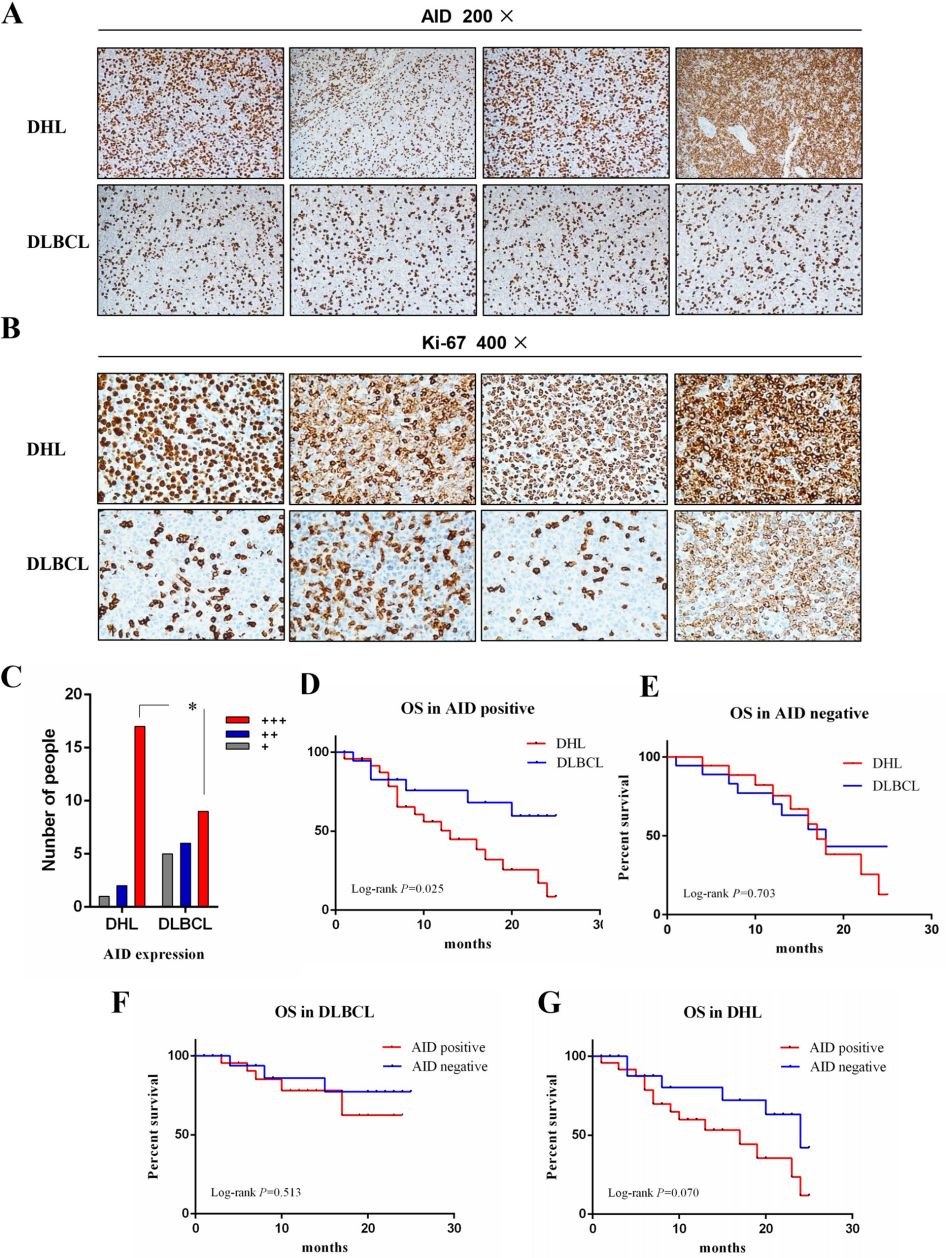
The clinical relationship between the expression of AID protein in DHL and DLBCL. (**A**, **B**) Expression of AID and Ki-67 was detected by immunohistochemistry in DHL and DLBCL. (**C**) The number of DHL and DLBCL patients with AID expression. (**D**, **E**) 2-year survival rate of DLH and DLBCL patients with AID protein positive and negative. (**F**) 2-year survival rate of patients with positive and negative DLBCL with AID protein. (**G**) 2-year survival rate of patients with positive and negative DHL with AID protein.

The relationship between AID expression and disease characteristics in 20 DHL and 20 DLBCL tumor cells are shown in Table 1. Immunohistochemistry was performed to determine the expression of AID and Ki-67 tumor tissues (Fig. 1A,B). The number of cases with AID positive cells number > 60% in DHL and DLBCL organization are 85% (17/20) and 45% (9/20), respectively. It is clearly that the number of cases of DHL group is significantly higher than that of DLBCL, with a statistically significant difference (*P* < 0.05) (Fig. 1C). We also analyzed the expression of some other related proteins in most samples, c-Myc in DHL 100% (20/20) and DLBCL 15% (3/20). DHL is more common in GCB immunophenotype, and Ki-67 is higher than DLBCL (Table 1). In addition, we also found that AID-positive DHL had higher IPI scores and Ann Arbor clinical staging than DLBCL (Table 2). We followed up the patients for 2 years, and the AID-positive DHL was worse than the AID-positive DLBCL OS, with a statistical difference (*P* = 0.025), (Fig. 1D), but there is no difference between AID-negative DHL and DLBCL (Fig. 1E). No significant difference in 2-year OS is observed for DLBCL patients with AID positive and negative (*P* = 0.513), (Fig. 1F). In addition, there is no statistical difference in 2-year OS between the DHL patients with AID positive and negative (*P* = 0.07), (Fig. 1G). However, the OS trend of DHL patients with AID positive is lower. These results indicate that AID is highly expressed in DHL and poor prognosis.

**Table 1:** Immunohistochemical characteristics in DHL and DLBCL.

Characteristic	DHL(*n* = 20)	DLBCL(*n* = 20)	*P* value
**Immunophenotype**			
GCB	19	10	0.001
Non-GCB	1	10	
**c-Myc**			
Negative	0	17	< 0.001
Positive	20	3	
**BCL-2**			
Negative	4	17	< 0.001
Positive	16	3	
**Ki-67**			
Negative (< 80%)	1	1	< 0.001
Positive (> 80%)	19	8	
AID	17	9	0.008

**Table 2:** Relationship of AID expression in DHL and DLBCL with clinicopathological features (n).

	DHL (n = 20)	DLBCL (n = 20)	*P* value
Age (years)	67.5	69.3	0.543
**Sex**			
Male	11	12	0.749
Female	9	8	
**IPI score**			
Low	0	3	0.189
Middle	5	5	
High	15	12	
**Ann Arbor**			
I–II	0	6	0.003
III	3	7	
IV	17	7	

### Enhanced CSR in DHL patients

**Figure 2:**
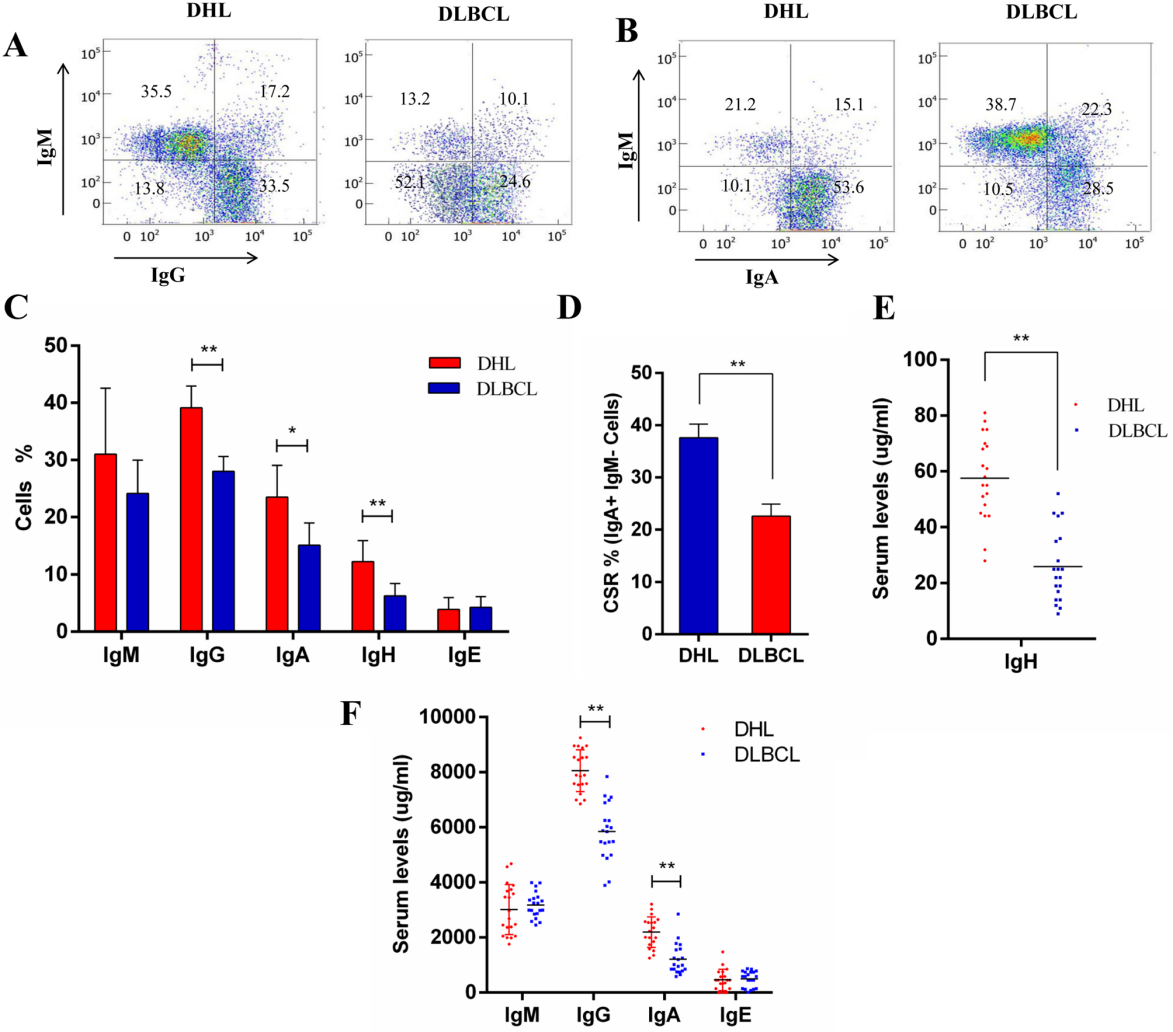
SCR is enhanced in patients with DHL. (**A**) Flow cytometric analysis for surface IgG expression in DHL and DLBCL peripheral blood. (**B**) Flow cytometric analysis for surface IgA expression in DHL and DLBCL peripheral blood. (**C**) Flow cytometry analyze the number of IgM, IgG, IgA and IgH cells in the peripheral blood of DHL and DLBCL patients (multiple *t* test). (**D**) Flow cytometry analyze the number of CSR cells in the peripheral blood of DHL and DLBCL patients (t test). (**E**) The IgH expression in DHL and DLBCL plasma. (**F**) Plasma IgM, IgG, IgA and IgH were detected by ELISA in DHL and DLBCL patients (*t* test). *P < 0.05, **P < 0.01.

AID induces the translocation of c-Myc/IgH in vivo, and CSR is required for antibody conversion of B cells. Therefore, the levels of IgM, IgA, IgG and IgH in DHL and DLBCL patients were measured to evaluate the level of CSR in vivo. Compared with patients with DLBCL, the level of IgG and IgA cells in the peripheral blood of patients with DHL increase (Fig. 2A,B). Specifically, the level of IgG, IgA and IgH cells in peripheral blood of patients with DHL are significantly enhanced (*P* < 0.05, Fig. 2C). In addition, CSR cells (IgA+ and IgM− cells) in patients with DHL are significantly higher than that of patients with DLBCL (*P* < 0.01, Fig. 2D). The same trend is observed in the plasma of patients in the two groups. The IgH expression in DHL plasma is significantly larger than that in DLBCL plasma (*P* < 0.01, Fig. 2E), while the IgG and IgA expression in DHL plasma are significantly increased compared with that in DLBCL (*P* < 0.01, Fig. 2F). Moreover, It can be concluded from the results that CSR is significantly enhanced in patients with DHL compared with that in patients with DLBCL.

### The enhancement of the expression of AID in DHL by CSR

**Figure 3:**
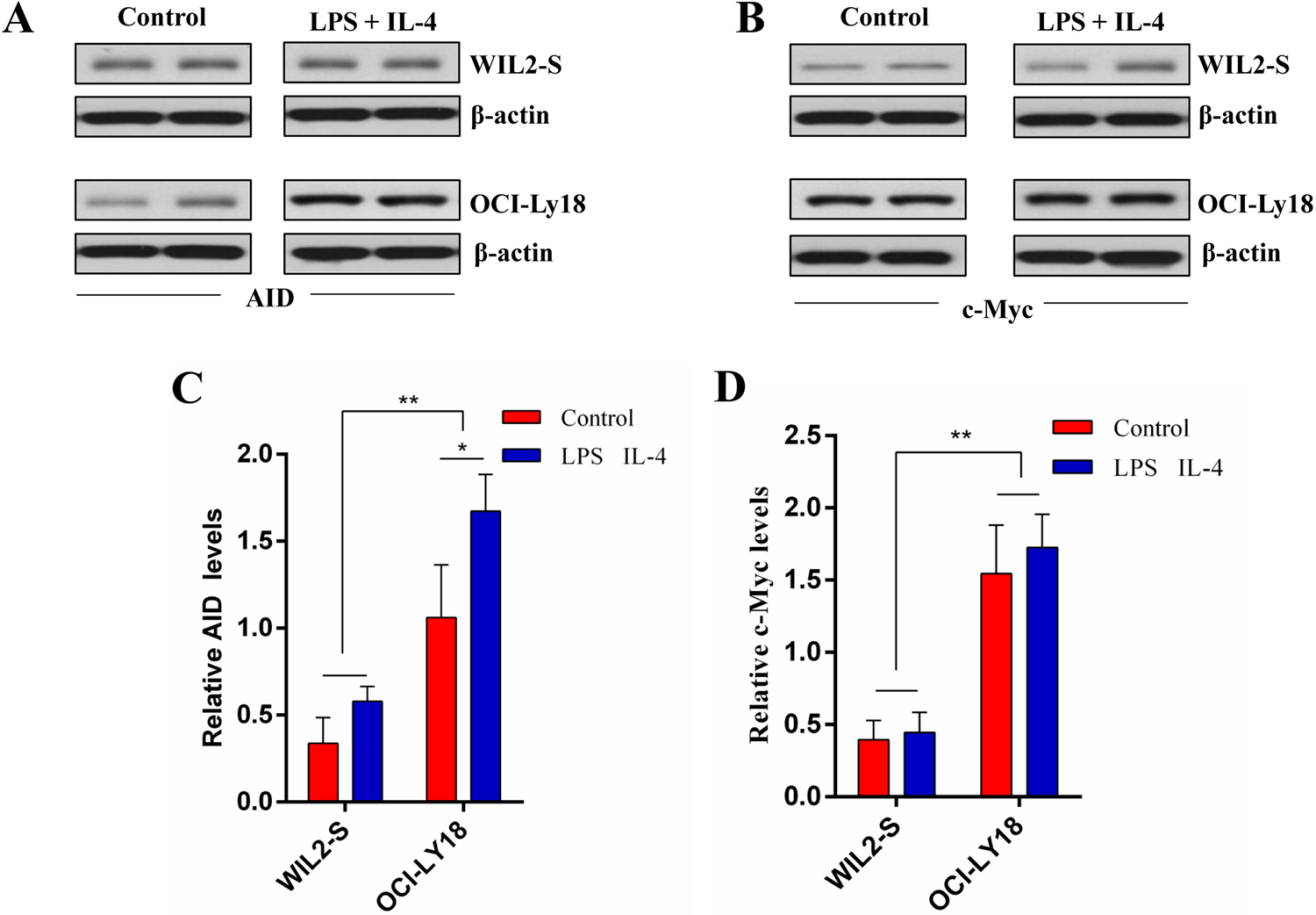
CSR promotes DHL cells AID expression. (**A**) Western blot of WIL2-S and OCI-LY18 cells AID expression. (**B**) Western blot of WIL2-S and OCI-LY18 cells c-Myc expression. (**C**) Summary of relative AID levels of n = 3 independent experiments. (**D**) Summary of relative c-Myc levels of n = 3 independent experiments. (**E**) RT-PCR detection of AID mRNA expression in WIL2-S and OCI-LY18 cells. (**F**) RT-PCR detection of c-Myc mRNA expression in WIL2-S and OCI-LY18 cells. All data represent mean ± SD from at least three independent experiments. Because the hard disk of the computer that saved the original data of the western blot experiment was damaged, only the electrophoresis after editing was saved. *P < 0.05, **P < 0.01 compared with control groups, respectively.

Normal B cells WIL2-S and DHL cells OCI-Ly18 were induced by LPS and IL-4. After the CSR of these two cells, the expressions of AID and c-Myc protein were detected by Western Blot (Fig. 3A,B). The expression of AID protein in OCI-Ly18 cells after CSR induced by LPS and IL-4 is significantly higher than that before CSR (*P* < 0.01, Fig. 3C). There is no difference in c-Myc protein of WIL2-S and OCI-Ly18 cells after CSR (*P* < 0.01, Fig. 3D). Meanwhile, the expression of AID and c-Myc protein in normal B cell WIL2-S are different from that in OCI-Ly18 cell (Fig. 3C,D). These results indicate that CSR enhances the expression of AID in DHL, while has little effect on the expression of c-Myc protein. In addition, we used RT-PCR to detect AID and c-Myc mRNA expression after CSR of the two cells. Normal human B cells WIL2-S did not enhance AID mRNA after induction of CSR in vitro (Fig. 3E). However, the expression of AID mRNA in double-strike lymphoma cells OCI-Ly18 induced CSR in vitro (*P* < 0.05, Fig. 3E). For c-Myc mRNA, expression of normal human B cells WIL2-S and DHL cells OCI-Ly18 did not increase after induction of CSR (Fig. 3F), but c-Myc mRNA of WIL2-S and OCI-Ly18 cells was significantly different (*P* < 0.05, Fig. 3F). In conclusion, the AID protein and gene levels of double-strike lymphoma cells OCI-Ly18 induced CSR in vitro significantly increased, indicating that CSR can enhance AID expression in DHL cells.

### CSR increased the translocation of DHL cell c-Myc/Igh

**Figure 4:**
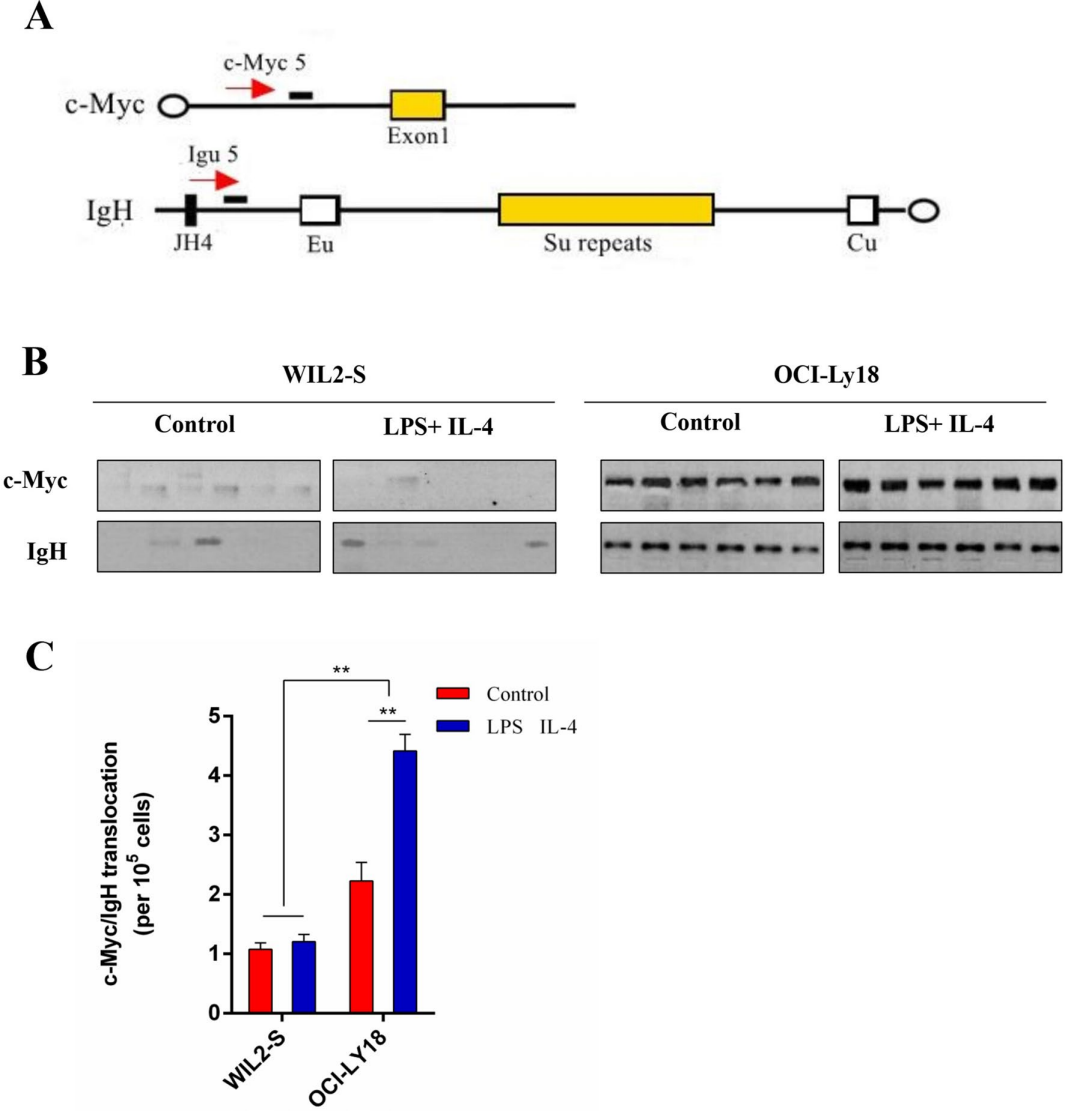
CSR promotes DHL cells Myc/Igh translocations. (**A**) Schematic for the Myc/Igh translocation assay. PCR amplification primers are represented by black arrows and Southern probes by gray bars. Closed circles denote centromeric locations on the chromosomes. (**B**) Representative translocation assay Southern blots with c-Myc and IgH probes are displayed. (**C**) Total translocation frequency summary from *n* = 3 independent experiments. The *P* value was determined with a two-tailed Fisher’s exact test.

AID induces the oncogenic c-Myc/Igh translocation by causing double strand break (DSB) at both Igh and c-Myc, with DSBs at c-Myc being rate limiting. However, whether CSR in DHL induces c-Myc/Igh translocation is still unclear. What we found is that the probability of c-Myc/Igh translocation in DHL can be enhanced by CSR. The frequency of translocation were determined by the PCR/Southern blot analysis as previously described (Fig. 4A)^15^. It is found that the c-Myc/Igh translocation of OCI-Ly18 is increased after CSR, while no increased translocation occur in normal B cell WIL2-S (Fig. 4B). The c-Myc/Igh translocation in OCI-Ly18 cells is significantly increased after CSR (*P* < 0.01), (Fig. 4C), while no difference of the c-Myc/Igh translocation in WIL2-S cells after CSR is found (Fig. 4C). There is a significant difference of Myc/Igh translocation between normal B cell WIL2-S and DHL cell OCI-Ly18 (*P* < 0.01), (Fig. 4C). The conclusion that CSR increases the translocation of c-Myc/Igh in DHL cell can be drawn.

## Discussion

DLBCL is the most common clinically diagnosed lymphoma, about half of which can be treated with standard chemoimmunotherapy^16^. The enhanced expression of c-Myc protein can be found in up to one-third of DLBCL cases, indicating that the change of c-Myc may be an important secondary conversion event^17^. Originally, DHL refers to patients with concurrent c-Myc and bcl-2 or bcl-6 gene translocations detected by FISH or standard cytogenetics. The DHL patients with the concurrent translocations of c-Myc and bcl-2 show clinical manifestations of strongly invasive and chemical-resistant, which exhibits very poor prognosis under the classic FISH definition. About one third of patients with DLBCL have long-term survival, which is significantly higher than that of DHL patients with c-Myc or bcl-2 translocation^18^. Due to the particularly poor outcomes of patients with c-Myc translocation shown in some retrospective studies, these patients were reclassified as high-grade lymphoma with c-Myc translocation and bcl-2 and/or bcl-6 translocation in the 2016 WHO classification of lymphatic tumors. These lymphomas are commonly referred to as double-blow lymphomas (DHL)^1^.

The main reason for the translocation of c-Myc in B cells is excessive CSR. To enhance the specificity and functionality of antibodies, immunoglobulin undergoes two DNA-changing events: somatic hypermutation (SHM) and CSR^19^. So far, AID is the only enzyme known to initiate SHM and CSR^20,21^, which is realized through programmed DNA damage of Ig locus. The double chain constant region of IgM is replaced with the other same type through DSB initiated by AID, finally realizing CSR^22^. The low affinity IgM antibodies with CSR and/or SHM impairment are produced in patients with AID mutations, which is known as a syndrome called high IgM immunodeficiency^23^. However, AID can also induce “off-target” DNA damage, leading to c-Myc/IgH DSB oncogenic chromosomal translocation^24^. Therefore, genome integrity can only be maintained by strictly controlling AID activities at different levels^25,26^. Generally, off-target mutations of AID and subsequent DSB and chromosomal translocations promote tumorigenesis, particularly for many types of B-cell lymphoma^27,28^. However, it has not been reported that whether c-Myc gene translocation is resulted from the overexpression of AID in DHL.

In this study, DHL and DLBCL clinical samples were collected. The differential expression of AID protein in the tissues of the two groups of patients were detected by Western-blot and immunohistochemistry. The results show that the expression of AID is significantly higher in patients with DHL than that in patients with DLBCL (Fig. 1), which indicates the significant correlation between AID and DHL. Numerous studies of Burkitt’s lymphoma with c-Myc translocation have found that c-Myc translocation is induced by AID^29,30^. In this study, it is suggested that AID is the main factor leading to c-Myc translocation in DHL patients, which is similar to Burkitt’s lymphoma. In addition, immunohistochemical results also show that most DHL belonged to the germinal center (GCB), with higher Ki-67 expression (Table 2), which is basically consistent with previous study^31^. Moreover, the 2-year OS of DHL and DLBCL patients were analyzed. Although the expression of AID is not significantly correlated with the 2-year OS of DHL and DLBCL (Fig. 1E,F), the trend of worse OS of DHL with positive AID is still observed. Thereby, it is suggested that AID is not only an important predictor of DHL diagnosis, but also an important indicator of poor prognosis. Based on these results, it can be speculated that AID positive large b-cell lymphoma is more prone to c-Myc gene translocation.

Higher grade transformation resulted from increased CSR that is induced by AID expression has been reported in several inert b-cell malignancies, including chronic lymphocytic leukemia and follicular lymphoma^10,14^. However, it has not been identified that c-Myc gene translocation of AID-positive DLBCL specimens increases compared with that of AID-negative DLBCL specimens in previous studies^32,33^. This study shows that a stronger CSR can be observed in patients with DHL than in patients with DLBCL (Fig. 2), which may be resulted from the enhancement of CSR caused by AID expression in DHL. To prove the relationship between AID and CSR in DHL, DHL cells OCI-Ly18 were cultured in vitro. It is found that the expression of AID protein was significantly increased when CSR is induced by LPS and IL-4, while the expression of c-Myc protein shows no significant difference (Fig. 3). In previous studies, CSR can directly lead to increased expression of AID protein, while no changes in c-Myc is observed. It is because in-pair DNA break is required for the abnormal connection of heterologous chromosomes^34^, while DNA has been damaged in mature b-cell malignant tumors^35^. However, our results showed the opposite result. When LPS and IL-4 were used to induce CSR of OCI-Ly18 cells, an increase of c-Myc translocation was observed (Fig. 4), which may be due to the fragile non-B-DNA to environmental factors (such as reactive oxygen intermediates) or the increased susceptibility of transcription/replication of related DNA damage^36^, or higher break levels of c-Myc or IgH or both^24^. In conclusion, our study reveals that AID is an independent risk factor in DHL, which may promote CSR process in vivo and ultimately lead to c-Myc translocation in DHL patients. Studies have proved that imatinib can be used as an AID inhibitor to reduce CSR in vivo^37^. It will provide a target for the treatment of DHL patients with c-Myc translocation by clarifying the role of AID in DHL.

## Materials and methods

### Patients

20 patients with DHL treated in the hematology department of Jinhua Hospital affiliated to Zhejiang University from January 2015 to February 2017 were included. All patients were identified by FISH as c-Myc accompanied with bcl-2 or bcl-6 translocation. The complete medical records, paraffin specimens of tumor tissues and peripheral blood of all patients were collected. Among the 20 patients, 11 were male and 9 were female, with the age range from 29 to 84 years. The clinical staging and classification of DHL are subject to 2016 NCCN clinical practice guidelines^38^. Twenty age- and sex-matched cases with classic DLBCL patients were referred as controls. This study was approved by the Medical Research Ethics Committee of Jinhua Hospital affiliated to Zhejiang University (JH2016-36), and informed consent were signed by all enrolled patients signed in accordance with the ethical standards of the institutional research committee and the Declaration of Helsinki.

### Cell lines and reagents

The WIL2-S and OCI-Ly18 were available from American Type Culture Collection (ATCC, Manassas, VA, USA), in which WIL2-S is normal human B lymphocyte and OCI-Ly18 is an EBV-negative DHL cell with the alteration of c-Myc/bcl2 gene^39^. Cells were cultured in RPMI-1640 medium with 10% heat-inactivated fetal bovine serum (FBS) in a humidified atmosphere of 95% air and 5% CO_2_ at 37 °C.

### Immunohistochemistry

Immunohistochemistry (IHC) staining was performed using Elivision plus two-step system (Maxim Biotech Inc, Fuzhou, China) for protein determination of paraffin-embedded sections. c-Myc and bcl-2 are important markers of DHL^40^. CD10, bcl-6 and MUM1 can be used to distinguish GCB or non-GCB^41^ and Ki-67 is used to judge the proliferation of tumor cells^42^. Paraffin-embedded tissues were sectioned (5 mm thick). Tissue sections were primarily stained with indicated antibodies for immunohistochemical analysis. Then biotinylated secondary antibodies detected the signal with DAB. Staining grading: no positive cells (−) were found in the whole section; the number of positive cell < 20% (+); the number of positive cells was 20–60% (++); and the number of positive cells was > 60% (+++).

### Flow cytometric analysis

Sheep anti-mouse fluorescent markers, including IgM, IgA, IgG1, IgG2, and IgG3 (all from BD Biosciences USA) were used in single-cell suspension. The stained cells were quantified by flow cytometer (D3130, ACEA Biosciences, China).

### Elisa

The CSR in DHL and DLBCL patients was evaluated by detecting the level of IgM, IgA, IgG and IgH in vivo^43^. The concentration of serum Ig in DHL and DLBCL patients were measured by the Procarta Multiplex Immunoassay (Thermo Fischer) according to the ELISA manufacturer's instructions. For ELISA, spectraplate-96 HB plates (PerkinElmer, Waltham Mass, USA) were used to measure the levels of specific IgM, IgA and IgG in two groups of patients. IgM, IgA, IgG and IgH was purchased from Santa Cruz Biotechnology, Inc. (Dallas, TX, USA).

### Western blot analysis

WIL2-S and OCI-Ly18 cell lines were cultured in a culture flask with the volume of 25 cm^2^. After the stabilization, lipopolysaccharide (LPS) and interleuk-4 (IL-4) were added to culture medium for 72 h to induce CRS process in vitro^44^, LPS and IL-4 purchased from Sangon Biotech, Inc.(Shanghai, China), we used LPS (25 mg/ml) and IL-4 (5 ng/ml) to co-culture with cells to mimic CSR in vitro. Mononuclear cells were obtained by RIPA lysis buffer (Cell Signaling Technology, Beverly, MA, USA) and centrifuged at 4 °C at 2136×*g* to obtain total protein. The protein concentration was measured and quantified, and the protein was boiled. The 5% concentrated gel and 12% separated gel were prepared, followed by the gel electrophoresis in sodium dodecyl benzene sulfonate. The membrane was transferred by wet method. 5% skim milk powder was sealed at room temperature for 1 h, and incubated with a primary antibody solution (rabbit anti-human AID and c-Myc polyclonal antibody with a dilution of 1:100) at 4 °C overnight. The secondary antibody solution was incubated at room temperature for 1–2 h the next day. The results were visualized with the ECL detecting kit (Biological Industries, Cromwell, CT, USA). AID and c-Myc primary antibodies were purchased from Abcam, Inc. (Cambridge, UK).

### Chromosome translocation assay

The translocation assay has been previously described^45,46^. Naive B cells were cultured with LPS and IL-4 for 72 h. Ficoll gradient removal of dead cells was performed, and 96 separate PCRs on genomic DNA from 10^5^ cells were performed with primers that amplify derivative c-Myc-IgH translocations. The primer sequence is shown in Table 3. Amplified translocations were confirmed by Southern blots with probes internal to the primers used in the PCR assay^46^. The experiment was performed twice independently, and the *P* value was calculated using a two-tailed Fisher's exact test.

**Table 3:** PCR primer sequences required for chromosome translocation determination.

First-stage of PCR for chromosome 12 translocation	
5-TGAGGACCAGAGAGGGATAAAAGAGAA-3	5-GGGGAGGGGGTGTCTCTATAATAAGA-3
First-stage of PCR for chromosome 15 translocation	
5-ACTATGCTATGGACTACTGGGGTCAAG-3	5-GTGAAAACCGACTGTGGCCCTGGAA-3
Second-stage of PCR for chromosome 12 translocation	
5-CACCCTGCTATTTCCTTGTTGCTAC-3	5-GACACCTCCCTTCTACACTCTAAACCG-3
Second-stage of PCR for chromosome 15 translocation	
5-CCTCAGTCACCGTCTCCTCAGGTA-3	5-GTGGAGGTGTATGGGGTGTAGAC-3
The first PCR of chromosome 12 translocation (upstream of switch)	
5-GGCAACTTCAAATTCATTAAACCACAT-3	5-GGGGAGGGGGTGTCTCTATAATAAGA-3
The first PCR of chromosome 12 translocation (upstream of switch)	
5-AAATGTGAGTGACCCAGACA ACG-3	5-GACACCTCCCTTCTACACTCTAAACCG-3

### Statistical analysis

Differences in clinical characteristics between groups were compared using independent samples t-tests for continuous variables, with logarithmic transformation of each individual value, and Fisher’s exact tests for categorical variables. Descriptive statistics were performed according to the distribution of variables. Date are shown as means ± SE. The Chi-square test was used for comparisons of qualitative variables between groups. All statistical analyses were performed with SPSS software (version 17.0, SPSS Inc, Chicago, IL, USA). *P < 0.05 and **P < 0.01 were considered significant.

## Supplementary information

Supplementary Information.

## Data Availability

Original data files are available upon a reasonable request.
