# Supplementary material for: Activation-induced cytidine deaminase overexpression in double-hit lymphoma: potential target for novel anticancer therapy
Source: Sci Rep. 2020 Aug 25;10:14164. doi: 10.1038/s41598-020-71058-y (PMC7447639; doi:10.1038/s41598-020-71058-y)
Supplement: Supplementary file 1 — Supplementary Information. [file 41598_2020_71058_MOESM1_ESM.docx]

Activation-induced cytidine deaminase overexpression in Double-Hit Lymphoma: potential target for novel anticancer therapy

Jingcheng Zhang^1,2^, Yifen Shi^3^, Mingzhe Zhao^2^,Huixian Hu^2^ and He Huang^1^
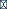


^1^Bone Marrow Transplantation Center, The First Affiliated Hospital, Zhejiang University School of Medicine;

^2^Department of Hematology, Jinhua Hospital of Zhejiang University(Jinhua Municipal Central Hospital);

^3^Department of Hematology, The First Affiliated Hospital of Wenzhou Medical University.

Correspondence to: He Huang, Email: huanghe@zju.edu.cn; Bone Marrow Transplantation Center, The First Affiliated Hospital, Zhejiang University School of Medicine; Hangzhuo, China; <Tel:0086-0571-87236703>; Fax:0751-87236562

**Supplementary Data**

**Supplementary Figure S1**


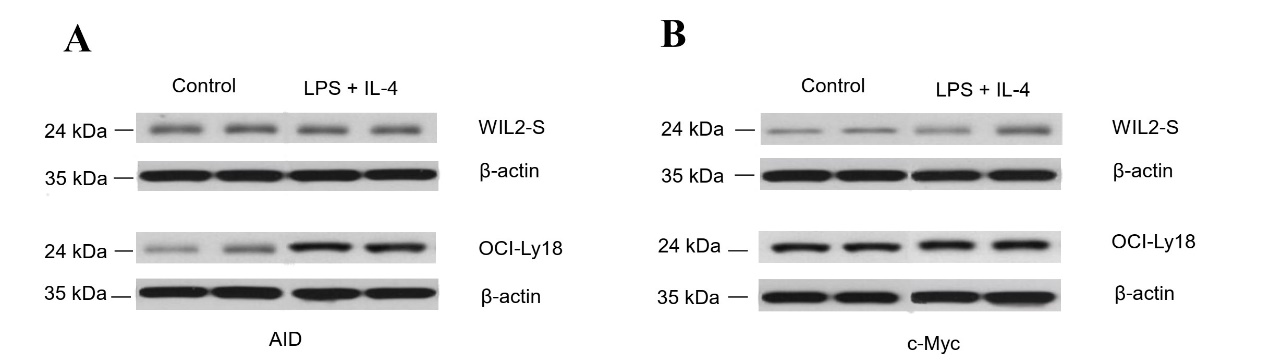


**Supplementary Fig. S1 Full size blots of the immunoblot detection.**

A: Full size blots of the immunoblot detection shown in Figure 3A. B: Full size blots of the immunoblot detection shown in Figure 3B.

**Supplementary** **Table S1**

| First-stage of PCR for chromosome 12 translocation | | | |  | | |
| --- | --- | --- | --- | --- | --- | --- |
| 5-TGAGGACCAGAGAGGGATAAAAGAGAA-3 | 5-GGGGAGGGGGTGTCTCTATAATAAGA-3 | | | | | |
| First-stage of PCR for chromosome 15 translocation | | | | | |  |
| 5-ACTATGCTATGGACTACTGGGGTCAAG-3 | 5-GTGAAAACCGACTGTGGCCCTGGAA-3 | | | | | |
| Second-stage of PCR for chromosome 12 translocation | | |  | | | |
| 5-CACCCTGCTATTTCCTTGTTGCTAC-3 | 5-GACACCTCCCTTCTACACTCTAAACCG-3 | | | | | |
| Second-stage of PCR for chromosome 15 translocation: | |  | | | | |
| 5-CCTCAGTCACCGTCTCCTCAGGTA-3 | 5-GTGGAGGTGTATGGGGTGTAGAC-3 | | | | | |
| The first PCR of chromosome 12 translocation (upstream of switch) | | | |  | | |
| 5-GGCAACTTCAAATTCATTAAACCACAT-3 | 5-GGGGAGGGGGTGTCTCTATAATAAGA-3 | | | | | |
| The first PCR of chromosome 12 translocation (upstream of switch) | | | | |  | |
| 5-AAATGTGAGTGACCCAGACA ACG-3 | 5-GACACCTCCCTTCTACACTCTAAACCG-3 | | | | | |

**Supplementary Table S1: PCR primer sequences required for chromosome translocation determination.**

**Supplementary Figure S2**

**
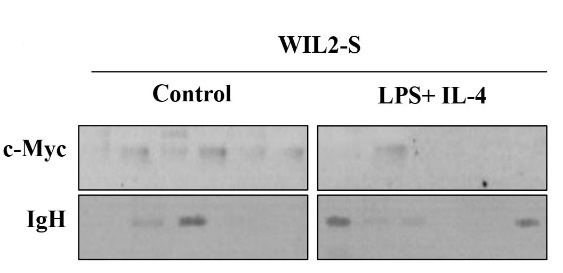
**


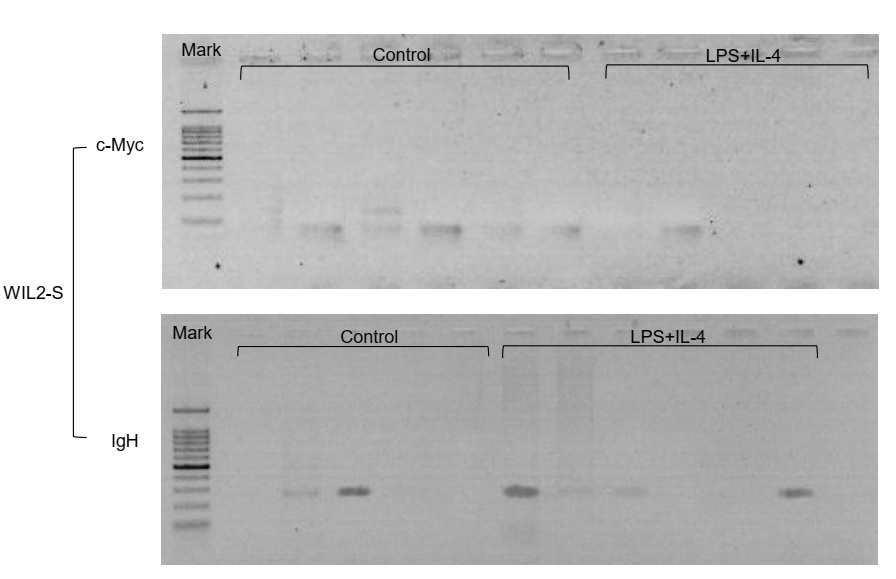


**
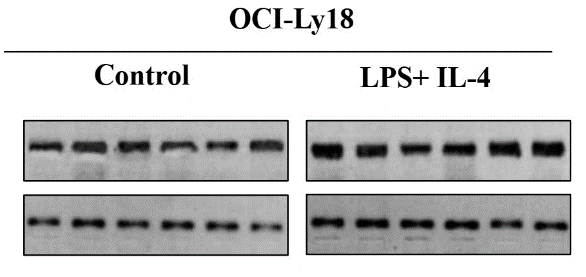

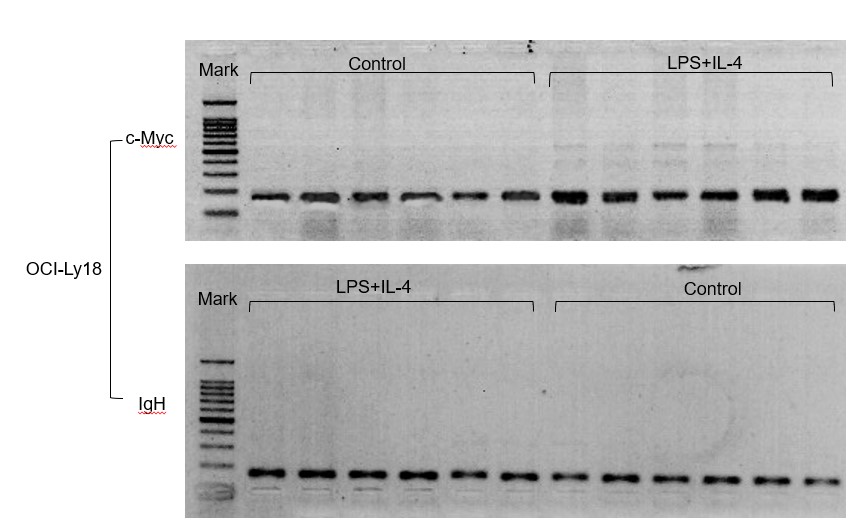
**

**Supplementary Figure S2 Full size** **PCR gel electrophoresis.**

Full size PCR gel electrophoresis shown in Figure 4B.
